# Six ways to foster community-engaged research during times of societal crises

**DOI:** 10.2217/cer-2020-0206

**Published:** 2020-10-30

**Authors:** Hillary A Edwards, Dwyan Y Monroe, C Daniel Mullins

**Affiliations:** 1 Department of Pharmaceutical Health Services Research, University of Maryland, Baltimore, MD 21201, USA; 2 Institute for Public Health Innovation, Washington, DC 20036, USA

**Keywords:** community-based participatory research (CBPR), community-engaged research (CEnR), community-academic partnerships, medical ethics, stakeholder engagement, trust

## Background

Historical mistreatment of black, indigenous and people of color (BIPOC) in healthcare and research has fueled mistrust and reluctance to participate in research [1–3]. In community-engaged research (CEnR) and patient-centered outcomes research (PCOR) studies, patients are valued as more than research subjects. When utilizing the 10-step framework for continuous patient engagement [4], patients and other stakeholders are active, equal and respected partners and their feedback is solicited and incorporated throughout the research process [5,6]. Patient engagement can lead to improved health outcomes [7], improved quality and patient safety [8], and impact healthcare costs and savings [9]. Incorporating diverse perspectives in the research process can make studies more inclusive, patient-centered, relevant and useful for informed decision-making. Patient engagement throughout and between research studies may improve relevance and uptake of research findings by patients and community healthcare providers and stakeholders [5].

What happens when research partnerships must respond to a societal crisis? In 2020, institutions froze or significantly altered their research operations in response to the coronavirus disease 2019 (COVID-19) pandemic [10]. Along with the difficulties of continuing or restarting research during a public health pandemic, CEnR partnerships must also confront the realities of distrust due to systemic racism, discrimination and violence [11] faced in BIPOC communities across the USA, once again magnified by the resurgence of the #blacklivesmatter movement [12]. While many CEnR best practices have been documented in the literature [7,13], in a time of crisis, people might be less likely to utilize best practices because it is challenging to do so. Moreover, for many BIPOC, the COVID-19 pandemic and the urgency to conduct research seemed like more of the same problems but a new disease.

To navigate authentic CEnR during a pandemic, a continuous research and community partnership must stand on a strong foundation of trust, transparency and accountability in order to traverse times of significant change and challenges. This article presents six best practices in CEnR and recommendations for continuous and authentic engagement when partnerships must decide how to move forward when facing systemic and acute crises.

The authors of this paper codeveloped the recommendations from their perspectives based on their partnership journey that has prevailed for over a decade. The first set of recommendations come from the community partner perspective and the second set come from the researcher perspective.

### Steps from the community perspective

#### 

##### Why research is necessary in ‘my’ community

Most researchers view me as monolithic – as a black female, a person with Type 2 diabetes, or someone from a faith-based organization. I belong to several communities and define my community as multicultural, complex and impacted by life’s injustices and social inequities. There are many benefits to BIPOC people participating in health research. Research findings that include subgroup analyses can help tailor results and recommendations to improve health equity and heal health disparities in BIPOC populations in the US. Despite the benefits, BIPOC participation in the US health research is under-represented [14,15]. A 2014 systematic review of barriers and facilitators identified five major barriers to health research participation, including mistrust, competing demands, concerns about unintended outcomes, lack of access to information and stigma [16]. The review also identified five facilitators for participation by nonwhite racial groups: cultural congruence, participant benefits, altruism, convenience and low-risk [16].

Community involvement in the development of the research protocol is critical for increasing BIPOC participation in research [17]. Community partners can define important issues to be evaluated in the study, such as survival, quality of life and access to care [6]. Partner involvement also can define the research question in a patient-centered voice, making it more easily understood and aligned with priorities of patients and participants.

##### I wish more researchers (& research teams) looked like me

The historical mistrust of medicine and research stems from research abuses typically were designed by white male doctors on BIPOC populations [18,19]. When researchers enter communities with paternalistic and messianic complexes, along with racial discordance, it preserves these negative legacies of distrust and mistreatment. Therefore, researchers must demonstrate cultural humility. Research teams must be diverse and inclusive of community members and actively express mutual respect and open communication beyond the scope of the project aims. Trust is not just about people but also about a community’s trust in institutions, which requires that these institutions are not only trusted but also trustworthy [13]. As with any partnership, trust builds over time. Codeveloping research with community partners, who may be community leaders or community-based organizations, assures the community at large that their voice is heard and prioritized as research operations occur. Peer interactions in research and cultural relevancy can be the best ice breaker and connector when working in communities that are at risk for inequities or doubtful of research intentions, particularly with ‘helicopter researcher’ [20]: when you fly in, gather your data and leave without further contact.

##### How I can help the research process when my community is at risk: codeveloping a plan

Speak with other community members to assess when it would be appropriate to restart research activities. Restarting activities too soon may lead to lower recruitment and negatively impact the reputation of the partnership. However, it is never ‘too early’ to discuss a timeline for readiness. As universities identify recovery plans for research operations, community partners must be part of the planning processes. Codeveloping a timeline to return to research with the community will demonstrate transparency and readiness for recovery. In addition, it is critical that community partners review and revise recruitment and retention plans determined prior to pandemics such as COVID-19 and determine if the prior methods are still appropriate.

### Steps from the researcher perspective

#### 

##### Give back to the community first & then ask for something: meet community members ‘where they are’ & continue to be there

Before you ask a community or one of its leaders to do something, contribute first to that community. Next, ask how you can help the community in addition to explaining how they can help your research. Honest communication requires active listening. It provides an opportunity to reset goals, boundaries and expectations. As a researcher, acknowledge the shift and actively listen to what your community partners are feeling, doing and needing. While an investigator may have received guidance about research operations, communicate these new parameters clearly to your community partners and answer questions about the research plan honestly. Provide a space and opportunity to check-in with your community partners consistently, not only regarding the research project but about their priorities and needs. Specific to COVID-19, research recovery discussions likely will come after addressing current concerns. As research institutions outline timelines for research recovery, ensure that your community partners are at the table when considering project restart time frames and processes.

There is an African proverb that goes, “If you want to go fast, go alone. If you want to go far, go together.” While research activities may be stopped, the relationship must continue forward. Meeting community partners where they are is not exclusive to geography, but also mentally, physically, emotionally and spiritually.

Sometimes, no action is needed; simply checking in with your partners demonstrates your commitment to the partnership, not only the research outcome. Successful research outcomes and community health outcomes can happen together.

##### Be transparent & share resources

Transparency reinforces trust and must be followed-up with accountability. Authentic partnership requires transparency in intention and action. Be clear about your intentions and accept that your participation may not be wanted. Check-in with partners and establish expectations on both ends for continuous communication to identify priority needs and appropriate timelines for restarting research. If limitations, elimination or changes in the research workplan occur, prioritize the messaging and get it out to the community. Once you have listened to the community’s priorities, share resources to evidence-based information that address those priorities. If you said you would return with new information or resources, follow through on your word. Radio silence does not foster community engagement and may damage your reputation with the community. Sharing resources is always important but particularly critical during the COVID-19 pandemic. Make sure that your community partners have what they need to continue to partner on your research project. Better yet, make sure that they have what they need to support community health and wellbeing.

##### Be a model for other researchers

CEnR and PCOR researchers should be actively engaged in more than their research; they also should be engaged in their partnerships and the activities that are important to their partners, especially during the COVID-19 pandemic. Coordinate public health messaging along with the information about the research enterprise to ensure streamlined communication gets to the community at large in a way that simultaneously advances scientific evidence and assure that evidence-based interventions are implemented to improve public health. In addition to talking with your community partners about your work, provide opportunities for community partners to consult with other investigators who are open to learning how to be better partners in research.

Reinforcing continuous engagement will demonstrate authentic and continuous commitment to CEnR, PCOR and community partnerships, leading to more impactful and more engaged research now and in the future. It also will improve public health and advance health equity.
